# Differential Effects of Novel Dopamine Reuptake Inhibitors on Interference With Long-Term Social Memory in Mice

**DOI:** 10.3389/fnbeh.2019.00063

**Published:** 2019-04-11

**Authors:** Judith Camats-Perna, Predrag Kalaba, Karl Ebner, Simone B. Sartori, Harish Vuyyuru, Nilima Y. Aher, Vladimir Dragačević, Nicolas Singewald, Mario Engelmann, Gert Lubec

**Affiliations:** ^1^AG Neuroendokrinologie und Verhalten, Institut für Biochemie und Zellbiologie, Otto-von-Guericke-Universität Magdeburg, Magdeburg, Germany; ^2^Department of Pharmaceutical Chemistry, Faculty of Life Sciences, University of Vienna, Vienna, Austria; ^3^Center for Molecular Biosciences Innsbruck (CMBI), Department of Pharmacology and Toxicology, Institute of Pharmacy, Leopold Franzens University Innsbruck, Innsbruck, Austria; ^4^Center for Behavioral Brain Sciences, Magdeburg, Germany; ^5^Department of Neuroproteomics, Paracelsus Medical University, Salzburg, Austria

**Keywords:** cognitive enhancement, social recognition memory, retroactive interference, aggression social interaction, dopamine transport inhibitor, long-term memory

## Abstract

In the laboratory, long-term social recognition memory (SRM) in mice is highly susceptible to proactive and retroactive interference. Here, we investigate the ability of novel designed dopamine (DA) re-uptake inhibitors (*rac*-CE-123 and *S*-CE-123) to block retroactive and proactive interference, respectively. Our data show that administration of *rac*-CE-123 30 min before learning blocks retroactive interference that has been experimentally induced at 3 h, but not at 6 h, post-learning. In contrast, *S-CE-123* treatment 30 min before learning blocked the induction of retroactive interference at 6 h, but not 3 h, post-learning. Administration of *S*-CE-123 failed to interfere with proactive interference at both 3 h and 6 h. Analysis of additional behavioral parameters collected during the memory task implies that the effects of the new DA re-uptake inhibitors on retroactive and proactive interference cannot easily be explained by non-specific effects on the animals’ general social behavior. Furthermore, we assessed the mechanisms of action of drugs using intracerebral *in vivo*-microdialysis technique. The results revealed that administration of *rac*-CE-123 and *S*-CE-123 dose-dependently increased DA release within the nucleus accumbens of freely behaving mice. Thus, the data from the present study suggests that the DA re-uptake inhibitors tested protect the consolidation of long-term social memory against interference for defined durations after learning. In addition, the data implies that DA signaling in distinct brain areas including the nucleus accumbens is involved in the consolidation of SRM in laboratory mice.

## Introduction

Social recognition memory (SRM) is the ability to distinguish between familiar and unfamiliar conspecific individuals (Thor and Holloway, 1982; Steckler et al., 1998). More than 100 years ago, Müller and Pilzecker postulated that information acquired during learning require some time to become long lasting memories and coined for this process the term “consolidation.” The same authors have introduced the concept of retroactive interference by determining that acquired information can be “displaced” by the amnesic effect of subsequent newly acquired information (Müller and Pilzecker, 1900). In contrast to retroactive interference, proactive interference is considered when the past learned event interferes with the acquisition/consolidation/retrieval of new information (Camats Perna and Engelmann, 2017).

Previous studies have shown that SRM is highly susceptible to manipulations aimed at producing retroactive and proactive interference (Dantzer et al., 1987; Engelmann, 2009). In the course of these studies, it was shown that the nature and timing of defined stimuli after and before learning, respectively, are the prominent factors to determine whether interference occurs. SRM experiments performed in mice demonstrated that after learning, retroactive interference could be observed up to 15 h and proactive interference can be observed up to 9 h. After learning, protein synthesis required for consolidation of both memory traces seems first to collide, then to compete, and finally overwrite each other in a time-dependent manner. After 18 h, both “memory traces” seem to dissociate and consolidate independently from each other (Engelmann, 2009).

The neuronal processing of stimuli acquired by defined sensory modalities may cause interference in SRM. Experiments investigating the basis for retroactive interference revealed that exposure to stimuli activating audition, taction, vision and olfaction up to 6 h after learning affect memory (Noack et al., 2010; Perna et al., 2015). It was also shown that stimuli which simultaneously activate different sensory modalities cause a robust interference when compared to stimuli that activate fewer sensory modalities: transient retrograde amnesia triggered by 1% isoflurane was able to block retroactive interference induced by an object stimulus, but had no effect when a conspecific stimulus animal was used to produce interference (Camats Perna and Engelmann, 2017). Thus, the manipulation of interference phenomena in SRM may both help to develop new pharmacological tools for the treatment of memory decline (“cognitive enhancers”) and provide new insight in the neuronal networks involved in the consolidation of this type of memory.

Modafinil is a wake-promoting drug which is used to treat sleep apnea, narcolepsy and shift work sleep disorders (Battleday and Brem, 2015; Kristofova et al., 2018). Recently, the synthesis and test in different behavioral paradigms of modafinil analogue 5-((benzhydrylsulfinyl)methyl)-thiazole (CE-123; Kalaba et al., 2017; Nikiforuk et al., 2017) was reported. CE-123 was structurally modified by substituting the carboxyl-amide moiety of modafinil with a heterocycle thiazole group attached to position five which may provide a high metabolic stability of CE-123. *In vitro* the racemate of CE-123 (*rac*-CE-123) blocks the dopamine transporter (DAT) with high specificity and no adverse side effects (Kalaba et al., 2017).

Pharmacokinetic studies showed that *rac*-CE-123 penetrates the blood-brain barrier and reaches its site of action in the brain within ~30 min after intraperitoneal administration in rats. Intraperitoneal administration of *rac*-CE-123 into Sprague-Dawley rats enhanced the acquisition and retrieval of memory in spatial hole-board task (Kristofova et al., 2018). It improved working memory in the radial arm maze and seems to modulate also the DA receptor *in vivo* (Kristofova et al., 2018). Further, *S*-CE-123 has also proven to enhance the cognitive flexibility without triggering unnecessary impulsive responding (Nikiforuk et al., 2017).

The present study was designed to assess the impact of CE-123 on the phenomenon of memory interference in SRM. In addition to the racemate, we used *S*-CE-123. The social discrimination task was performed in mice, and two different time points after the 1st sampling (3 and 6 h) were selected to evaluate possible effects on retroactive or proactive interference during SRM consolidation. Further, additional parameters were monitored during the behavioral tests to allow a first identification of possible behavioral side effects of the treatment that might have affected the behavioral readout interpreted as “memory.”

In addition, we used microdialysis to investigate the effects of a single systemic administration of *rac*-CE-123 and *S*-CE-123 on extracellular DA levels in the mouse nucleus accumbens. Previous studies have shown that this brain area might be involved in the correct processing of short-term SRM in rats (Ploeger et al., 1991).

## Materials and Methods

### Animals and Housing Conditions

For behavioral testing, adult male C57BL/6JOlaHsd mice (Harlan-Winkelmann, Borchen, Germany) with an age group of 9–16 weeks were used as experimental subjects. If not stated otherwise, they were housed in groups of five per cage (size: 20 × 37 × 15 cm) for at least 1 week before starting the experiments under standard laboratory conditions (temperature 22 ± 1°C, humidity 60 ± 5% with a 12:12 h light-dark cycle lights on: 07:00 h). Stimulus animals were C57BL/6JOlaHsd mice of both sexes with an age of 25–35 days. For microdialysis experiments, adult male C57BL/6J mice were used. These animals were kept under similar conditions and experiments starting at 08:00–08:30 h. All experimental manipulations were approved by the Committee on Animal Health and Care of the local governmental body (Regierungspräsidium, Halle, registered and approved: 42502-2-1365 UniMD; microdialysis procedures were approved by the Austrian Animal Experimentation Ethics Board; Bundesministerium für Wissenschaft Forschung und Wirtschaft, Kommission für Tierversuchsangelegenheiten) and performed in strict compliance with the EEC recommendations for the care and use of laboratory animals (2010/63/EU).

### Behavioral Procedure

**Figure 1:**
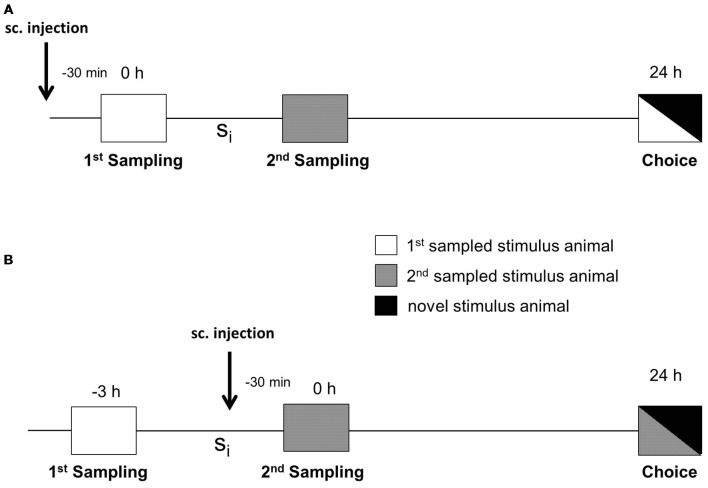
Experimental protocol for testing the effects of the defined substances on interference in social recognition memory (SRM). **(A)** Subcutaneous (sc) injection was performed 30 min before the 1st sampling to measure the impact of administered substances on retroactive interference during choice. **(B)** Subcutaneous injection was performed 30 min before the 2nd sampling to measure the effect of administered compounds on proactive interference. The two samplings were separated by a defined sampling interval (S_i_). Choice took place either 24 h after the 1st sampling, when testing retroactive interference **(A)**, or 24 h after the 2nd sampling when testing proactive interference **(B)**.

The social discrimination test performed has been described in detail in Engelmann et al. (2011). In brief, experimental subjects were separated 2 h before starting the session by transferring them to small cages with fresh bedding. The test procedure consisted of two sampling sessions (4 min each) and one choice session (4 min) performed in the adult’s cage under dimmed lighting conditions (during the light phase, i.e., between 8:00 and 15:00 h). During the 1st sampling, a given stimulus animal was exposed to the experimental subject and the behavior of the latter was monitored by pressing the pre-set key on a laptop by a trained observer unaware of the experimental subjects’ treatment. The stimulus animal was then removed and kept individually in a new cage with food and water *ad libitum*. As illustrated in Figure 1, after a defined sampling interval (S_i_), a second, previously not encountered stimulus animal was presented for 4 min to the experimental subject during the 2nd sampling session. To measure retroactive interference, during retrieval (choice), the stimulus animal encountered during the 1st sampling was presented to the experimental subject together with a novel stimulus animal 24 h after the 1st sampling. To measure the proactive interference, during the choice session, the 2nd sampled stimulus animal was presented to the adult together with a novel stimulus animal. Significant longer investigation of the novel stimulus animal compared to the already encountered stimulus animal during choice was taken as evidence for an intact recognition memory (Thor and Holloway, 1982; Engelmann et al., 2011). Earlier studies revealed that the consolidation of long-term SRM corresponds to two phases of anisomycin sensitivity with a gap at 3 h after sampling (Richter et al., 2005). We used both an S_i_ of 3 h (in the gap) and 6 h (after the gap) for our studies.

In addition to the investigation duration also the latency between the introduction of the stimulus animal in the experimental subject’s cage and the first approach of the experimental subject towards the stimulus animal was monitored. Also, the duration of aggressive and sexual behavior of the experimental subject towards the given stimulus animal during the 1st and 2nd sampling was monitored.

### Drug Treatment

The following drugs were used in the present study: *rac*-CE-123 = 5-((benzhydrylsulfinyl)methyl)-thiazole (Kristofova et al., 2018) and *S*-CE-123 = S-5-((benzhydrylsulfinyl)methyl)thiazole (Nikiforuk et al., 2017). The dosage of the drugs and the time point of administration were selected according to previous studies in which it was shown that 10 mg of the drugs per kg body weight administered 30 min before testing produces significant learning and memory effects without causing detectable undesired side effects in rats (Nikiforuk et al., 2017; Kristofova et al., 2018). The drugs were dissolved in 1% DMSO and 3.3% Tween 80 diluted in 0.9% NaCl. The solution contained 1 mg/ml and the dosage administered was 10 mg/kg body weight for all drugs. Vehicle contained the solvent (1% DMSO and 3.3% Tween 80 diluted in 0.9% NaCl) only. The experiments were performed in a double-blind cross-over design. Thus, all animals received both vehicle and the given drug in a random order. The code was broken after the end of the behavioral experiments when the data was assigned to each treatment conditions and finally analyzed.

For retroactive interference, vehicle or drugs were administered subcutaneously (sc) 30 min before the 1st sampling session (Figure 1A). For proactive interference, sc administration was performed 30 min before the 2nd sampling session (Figure 1B). The testing of the effects of *S*-CE-123 on proactive vs. retroactive interference was incorporated to get a first insight into the timing and possible interactions of potentially DA signaling for early or late stabilization of an SRM trace.

### Microdialysis

For the preparation of the microdialysis experiment, mice were anesthetized (5 mg/kg xylazine, 80 mg/kg ketamine, i.p., isoflurane) and placed in a stereotaxic frame (David Kopf Instruments, Tujunga, CA, USA). A guide cannula (MAB 4.15.IC, Microbiotech, Stockholm, Sweden; o.d. 0.48 mm) was implanted unilaterally 1 mm above the right nucleus accumbens (A/P = +1.0 mm, L/M = +0.8 mm, D/V = −3.6 mm) according to the mouse brain atlas by Franklin and Paxinos (2007) and fixed to the skull with dental acrylic cement and two stainless steel screws. Animals received buprenorphine (5 mg/kg, sc) and an analgesic *via* the drinking water (Meloxicam, 5 mg/kg, for 3 days) for post-surgery care and were housed individually. The evening before the microdialysis experiment, mice were shortly anesthetized with isoflurane and a microdialysis probe (MAB 4.15.1, Microbiotech, Stockholm, Sweden) with a molecular cutoff of 6 kDa (o.d. 0.2 mm, PES membrane 1 mm of length) was inserted into the guide cannula of mice reaching into the nucleus accumbens. The probe was connected to a CMA/Microdialysis Syringe pump (CMA-4004) and constantly superfused with sterile artificial cerebrospinal fluid (aCSF; NaCl 140 mM, KCl 3.0 mM, CaCl_2_ 1.25 mM, MgCl_2_ 1.0 mM and Na_2_HPO_4_ 1.2 mM and NaH_2_PO_4_ 0.3 mM; pH 7.4) at a flow rate of 0.5 μl/min. On the day of experiment, superperfusion rate was set to 1.0 μl/min and after 2 h of equilibration sequential microdialysis fractions were collected every 20 min into ice-cooled microtubes containing 6 μL of an antioxidative mixture (100 mM acetic acid, 0.27 mM Na_2_EDTA and 12.5 μM ascorbic acid), vortexed and stored at −80°C until further analysis. After three baseline samples (collected from −60 to 0 min), vehicle or drugs (10 mg/kg, sc) were administered, and six samples were collected. Subsequently, vehicle or drugs were administered in a higher concentration (100 mg/kg, sc) and another six microdialysates were collected. For the last two dialysates aCSF containing 100 mM KCl was used as a positive control to elicit local depolarization in order to confirm the functionality of the system. At the end of the experiment, mice were euthanized by an overdose of thiopental and brains were removed for histological verification of the placement of microdialysis probes. Data were only used from subjects with correct probe displacement (see Figure 5A).

**Figure 2:**
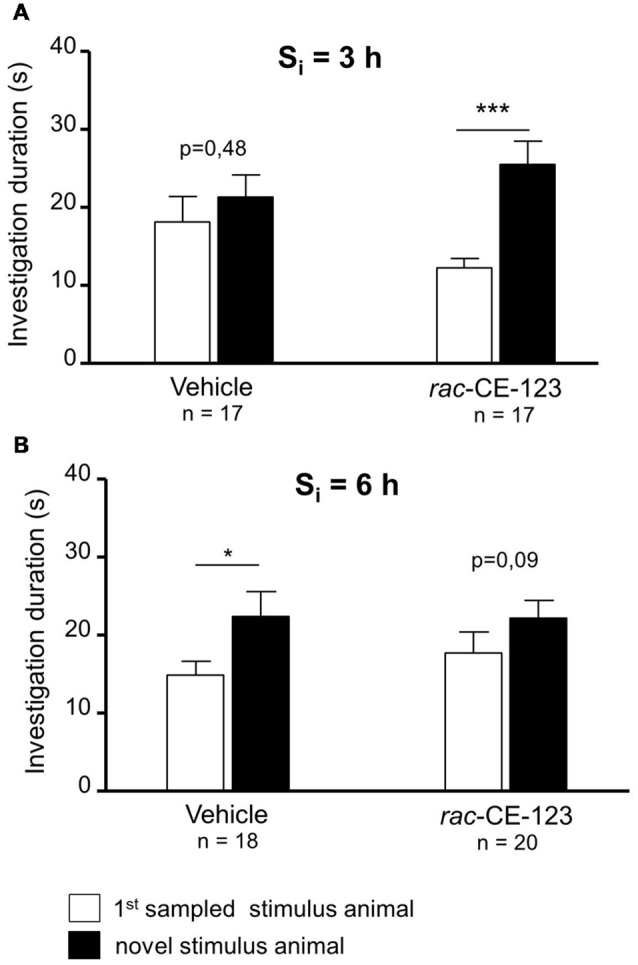
Effect of subcutaneous injection of vehicle or *rac*-CE-123 on retroactive interference at S_i_ = 3 h **(A)** and 6 h **(B)** on social investigation. Recognition memory was tested during choice by exposing the stimulus animal presented during the 1st sampling (1st S) together with a novel stimulus animal mouse 24 h after the 1st sampling. *p < 0.05 and ***p < 0.01 paired Student’s *t*-test.

**Figure 3:**
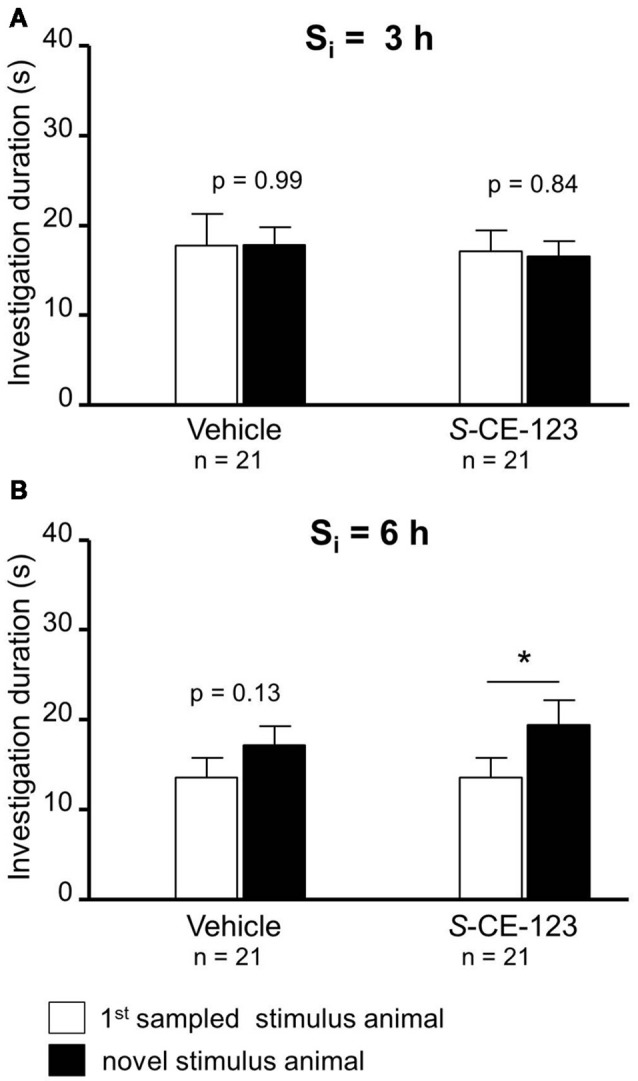
Effect of subcutaneous injection of vehicle or *S*-CE-123 on retroactive interference at S_i_ 3 = h **(A)** and 6 h **(B)** on social investigation. Recognition memory was tested during choice by exposing the stimulus animal presented during the 1st sampling (1st S) together with a novel stimulus animal 24 h after the 1st sampling. *p < 0.05 paired Student’s *t*-test.

**Figure 4:**
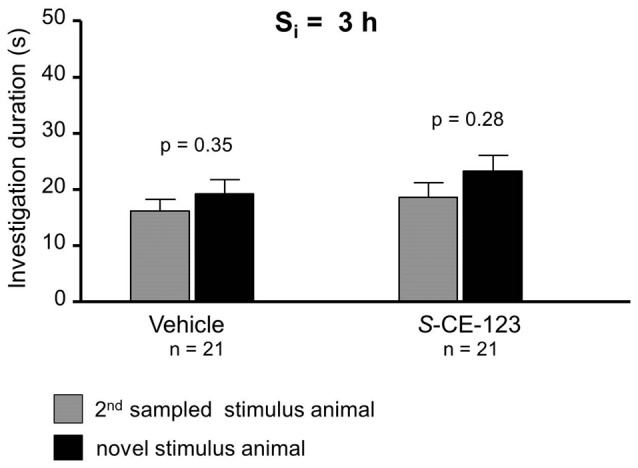
Effect of *S*-CE-123 on proactive interference at a S_i_ 3 h. Recognition memory of the experimental subjects treated with *S*-CE-123 was tested during choice by exposing the stimulus animal presented during the 2nd sampling (2nd S) together with a novel stimulus animal.

**Figure 5:**
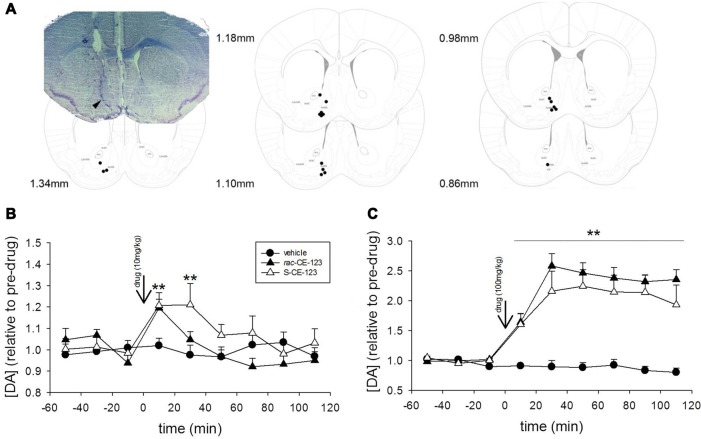
Microdialysis experiments. Coronal section diagrams modified from Franklin and Paxinos (2007) and a representative photomicrograph illustrating the reconstructed tip of the probe placement and the track left by the microdialysis probe in the brain tissue, respectively in the nucleus accumbens **(A)**. Effect of subcutaneous injection of vehicle or a low dose (10 mg/kg, **B**) and high dose (100 mg/kg, **C**) of *rac*-CE-123 or *S*-CE-123 on DA levels in dialysates from the nucleus accumbens of mice. Dopamine values in microdialysates are shown as changes in basal DA values, calculated as the mean of three consecutive samples immediately preceding the drug or vehicle injection. Data are expressed as mean ± SEM. *n* = 6 per group; **p < 0.01 vs. respective value in the vehicle-treated controls (two-way ANOVA followed by Fisher’s *post hoc* test).

### Analysis of Dopamine

Dopamine was analyzed in 5 μl microdialysate fractions by high-performance liquid chromatography (HPLC) with electrochemical detection. The HPLC system consisted of a Shimadzu (Kyoto, Japan) system controller (CBM-20A), degassing unit (DGU-20A3R) and micro HPLC pump (LC-20ADXR) operated at a flow rate of 55 μl/min. The mobile phase consisted of 8% (v/v) methanol, 50 mM phosphoric acid, 50 mM citric acid, 2.36 mM octane-sulfonic acid, 0.1 mM Na_2_EDTA at a pH of 5.6. Samples were injected *via* a SIL-20ACXR autosampler (Shimadzu, Japan) and separated on a C18 reversed-phase column (NeuroSep 105; 50 mm × 1.0 mm i.d.; 3 μM spherical particles; Antec, Zoeterwoude, Netherlands). The HPLC system was coupled to the DECADE II electrochemical detector (Antec SenCell, 2 mm glassy carbon working electrode, Ag/AgCl reference electrode, Antec Zoeterwoude, Netherlands). The column and detector cell were maintained at 35°C by a column oven as part of the electrochemical detector. The applied potential was set to +460 mV vs. reference electrode and was adjusted to a detection range of 100 pA/V with a filter frequency setting of 0.01 Hz. Substance amounts which yielded a detector signal corresponding to three times noise level were considered at detection limit. This allowed for the measurement of DA with a sensitivity of 0.25 fmol/5 μl sample. Instrument control and data acquisition were carried out by Lab Solution chromatography software (LabSolution CS, Shimadzu, Japan). Calibration curves were constructed in the range of 50 pM to 1 nM (0.25–5 fmol of DA injected) and were consistently linear with correlation coefficients higher than 0.999.

### Statistics

Data are presented as mean + SEM. Statistical analysis of the behavioral data was performed by GraphPad Prism 6.05 (GraphPad Software, San Diego, CA, USA). Data obtained from the social discrimination experiments were analyzed using the paired Student’s *t-*test. The additional behavioral parameters (latency from the experimental subject to investigate the stimulus animal after its introduction and duration of aggressive behavior) were analyzed using one-way ANOVA. For the microdialysis experiments, the DA content in each 20 min-microdialysate was expressed as a relative value to the mean content rates of the three samples preceding the administration of the drug or vehicle. Statistical analysis was carried out with Statistica Software v9 [StatSoft (Europe) GmbH, Hamburg, Germany] using two-way ANOVA for repeated measures followed by Fisher’s test. *P* < 0.05 was considered to be statistically significant.

## Results

Investigation durations measured during the 1st and 2nd sampling are presented in Table 1. When tested under vehicle conditions, in total two animals (for *rac*-CE-123 at a S_i_ = 6 h) had to be excluded from the analysis of the treatment conditions because the investigation duration during the 1st or 2nd sampling was <1 s and, thus, it is unreliable to assume that sufficient information was acquired for a successful recognition and interference, respectively. The data of the remaining animals show that the average investigation duration during both sampling sessions was sufficient to acquire the important information essential to establish long-term SRM and to produce an interference, respectively (Engelmann et al., 2011).

**Table 1:** Investigation durations (means + SEM) during the 1st and 2nd sampling of the animals presented in Figures 2–4.

Corresponding Figure	S_i_ (type of interference)	Treatment	1st sampling	2nd sampling	*n*
2A	3 h (retroactive)	Vehicle	23.53 + 4.59	16.34 + 3.02	17
		*rac*-CE-123	26.20 + 3.34	15.83 + 4.19	17
2B	6 h (retroactive)	Vehicle	24.02 + 3.70	14.01 + 2.73	18
		*rac*-CE-123	27.09 + 3.46	15.53 + 2.07	20
3A	3 h (retroactive)	Vehicle	21.41 + 3.00	17.50 + 2.70	21
		*S*-CE-123	27.79 + 2.56	17.12 + 2.40	21
3B	6 h (retroactive)	Vehicle	26.04 + 3.82	28.68 + 4.82	21
		*S*-CE-123	25.35 + 4.60	27.30 + 4.15	21
4	3 h (proactive)	Vehicle	30.78 + 2.80	30.78 + 2.80	21
		*S*-CE-123	34.17 + 4.50	28.37 + 4.38	21

*It shows the investigation duration of experimental subjects towards presented stimulus animals during the 1st and 2nd sampling sessions of retroactive and proactive interference experiments (see Figures 1–4). *n* = number of animals per group*.

When retroactive interference was introduced at a S_i_ = 3 h, *rac-*CE-123-, but not vehicle-administered experimental subjects showed significantly longer investigation durations towards the novel stimulus animal than towards the 1st sampled stimulus animal during choice (Figure 2A; paired Student’s *t*-test; vehicle: *t* = 0.73, *p* = 0.475; drug: *t* = 4.02, *p* = 0.001). At a S_i_ of 6 h, *rac*-CE-123-treatment failed to significantly affect the investigation durations during choice (Figure 2B; paired Student’s *t*-test: *t* = 1.81, *p* = 0.087). However, vehicle administered experimental subjects investigated the novel stimulus animal significantly longer than the 1st sampled stimulus animal during choice (Figure 2B; paired Student’s *t*-test: *t* = 2.15, *p* = 0.047).

Neither administration of *S*-CE-123 nor that of vehicle caused a significant difference in the investigation of the 1st sampled and the novel stimulus animal during choice at a S_i_ = 3 h (Figure 3A; paired Student’s *t*-test; vehicle: *t* = 0.01, *p* = 0.993; drug: *t* = 0.21, *p* = 0.838). If the same drug was administered at a S_i_ = 6 h, during the choice session experimental subjects investigated the novel stimulus animal longer than the 1st sampled stimulus animal (Figure 3B; paired Student’s *t*-test: *t* = 2.54, *p* = 0.020). Vehicle treatment failed to affect significantly the investigation durations (paired Student’s *t*-test: *t* = 1.57, *p* = 0.131).

When testing proactive interference and introduced at S_i_ of 3 h, neither vehicle (Figure 4; paired Student’s *t*-test: *t* = 0.96, *p* = 0.348) nor *S*-CE-123 (Figure 4; paired Student’s *t*-test: *t* = 1.11, *p* = 0.279) showed a significant difference in investigation duration between 1st sampled stimulus animal and novel stimulus animal during the choice session.

The data collected from the additional parameters monitored during the behavioral testing are shown in Table 2. Using S_i_ = 3 h, no significant effects on any of the additional behavioral parameters monitored were detected (*via* ANOVA), independently upon the administered substance (vehicle or drug). In contrast, ANOVA statistical test revealed a significant effect on the latency to start investigating the stimulus animal during the 2nd sampling at a S_i_ = 6 h in case of vehicle treatment only, for *rac*-CE-123 and its respective vehicle treatment only. Subsequent analysis *via* Student’s *t*-test failed to detect significant differences between the 1st and 2nd sampling under a vehicle and the respective drug treatment (data not shown). Thus, the differences detected *via* ANOVA resulted from different values measured during the 1st sampling vs. the 2nd sampling and did not reflect a specific treatment effect.

**Table 2:** Experimental subject’s latency to investigate and duration of aggressive behavior (means in seconds + SEM) towards the presented stimulus animal measured during the 1st and 2nd sampling, respectively.

S_i_	Parameter	1st sampling		2nd sampling		ANOVA
		Vehicle	Drug	Vehicle	Drug	
*rac-*CE-123 (retroactive)
3 h	Latency	5.50 + 2.08	3.45 + 0.85	28.13 + 11.07	18.16 + 11.21	*F*_(3,64)_ = 2.11; *p* = 0.11
	Aggression	3.25 + 0.71	3.80 + 1.14	2.19 + 1.00	5.14 + 1.42	*F*_(3,64)_ = 1.25; *p* = 0.30
6 h	Latency	8.62 + 2.38	2.46 + 0.44	21.90 + 7.39	10.41 + 1.79	*F*_(3,72)_ = 4.49; *P* < 0.01
	Aggression	2.59 + 1.04	1.62 + 0.50	1.90 + 0.78	1.01 + 0.35	*F*_(3,72)_ = 0.90; *p* = 0.44
*S*-CE-123 (retroactive)
3 h	Latency	2.62 + 0.34	3.13 + 0.55	4.47 + 1.24	6.44 + 1.79	*F*_(3,80)_ = 2.25; *p* = 0.09
	Aggression	2.05 + 0.40	1.57 + 0.42	2.57 + 0.73	1.90 + 0.47	*F*_(3,80)_ = 0.63; *p* = 0.63
6 h	Latency	3.38 + 1.16	3.85 + 0.70	4.66 + 0.98	2.53 + 0.49	*F*_(3,80)_ = 1.05; *p* = 0.38
	Aggression	1.65 + 0.37	3.79 + 1.25	4.81 + 1.10	5.19 + 1.38	*F*_(3,80)_ = 2.10; *p* = 0.11
*S*-CE-123 (proactive)
3 h	Latency	4.05 + 1.07	5.03 + 1.85	3.48 + 0.71	4.60 + 1.23	*F*_(3,80)_ = 0.27; *p* = 0.84
	Aggression	2.30 + 0.72	1.96 + 0.54	4.32 + 0.69	4.96 + 1.13	*F*_(3,80)_ = 2.92; *p* = 0.03

*Experimental subjects were treated with either *rac-*CE-123 or *S*-CE-123 30 min before the 1st sampling (when testing retroactive interference) or the 2nd sampling (when testing proactive interference) and subsequently tested as illustrated in Figures 1A,B). The ANOVA results refer to the values of the sample line*.

A representative example of a correct placement of the microdialysis probe in the nucelus accumbens is shown in Figure 5A. DA levels in microdialysates reached stable baseline values of 0.54 ± 0.08 fmol/5 μl sample. No significant differences in basal dialysate concentrations of DA between vehicle-treated control group and drug treatment groups were found. Drug treatment significantly affected DA levels over time at both the low (drug × time interaction: *F*_(16,120)_ = 1.949, *p* < 0.05) and high doses (drug × time interaction: *F*_(16,120)_ = 11.418, *p* < 0.001). While vehicle treatment failed to alter the DA concentrations, sc injections of both *rac*-CE-123 and *S*-CE-123 increased the concentrations of DA in the microdialysates. Specifically, the administration of 10 mg/kg of *rac*-CE-123 and *S-*CE-123 caused a moderate increase in DA concentrations for at least 20 min and 40 min, respectively, and returned to baseline levels within the subsequent 40–60 min (Figure 5B). The high dose (100 mg/kg) of both *rac*-CE-123 and of *S*-CE-123 caused a maximum increase in the DA concentration within 40 min (*p* < 0.01) after injections and remained elevated throughout the whole sampling period (Figure 5C).

## Discussion

The present study was designed to investigate the impact of recently synthesized and potentially cognitive enhancing drugs on retroactive and proactive interference of SRM. For this purpose, mice injected sc with either *rac-*CE-123 or *S*-CE-123 were tested in the social discrimination task. Treatment with *rac*-CE-123 blocked the otherwise seen retroactive interference at 3 h after the 1st sampling (Figure 2). However, if the S_i_ between both samplings was 6 h, both drugs failed to affect retroactive interference (Figure 2). Surprisingly, when treated with a vehicle at a S_i_ = 6 h during the choice session, experimental subjects explored the novel stimulus animal significantly longer than the 1st sampled one (Figure 2B). At first glance, this may indicate that the vehicle treatment has blocked interference. The investigation duration during the 2nd sampling was similar to the 1st sampling (Table 1) with the ANOVA detecting an increased latency to investigate an increased aggression (Table 2). A similar effect of vehicle treatment on investigation durations during choice, however, is not observed in the other groups tested in the present study (Figures 2, 3) and numberous own unpublished observations with other drugs including modafinil analogues applied under otherwise identical conditions and solvent. Thus, we propose to consider this as an extraordinary outliner, which, nevertheless, will be the focus of further investigations.

With respect to the observed blockage of retroactive interference, administration of *rac-*CE-123 seems to affect the information processing linked to the 1st sampling in a way that it becomes insensitive to a potential interference impact within <6 h after learning. It is well known that blocking the DAT activity by psychostimulants prevents the re-uptake of DA and increases the extra-synaptic concentration of DA in the brain (Kuhar et al., 1991; Li et al., 1996). Indeed, our microdialysis experiments revealed a significant increase of extracellular DA levels in dialysates collected from the nucleus accumbens after sc administration of both *rac*-CE-123 and *S*-CE-123. Both drugs increased the DA concentration in the mcirodialysates in a dose- and time-dependent manner. Notably, at the concentration of 10 mg/kg the maximal response occurred within the first 20 min followed by a gradual decrease within the next 1–2 h (Figure 5B). The drug-induced release profiles suggest that increased DA levels are to be expected for at least 30–40 min after the sc injection and thus during sampling in the social discrimination task. The data demonstrating a rapid and long-lasting stimulatory effects of novel modafinil analogues *rac*-CE-123 and *S*-CE-123 on extracellular DA levels in distinct brain areas including the nucleus accumbens are in line with previous microdialysis studies using modafinil and/or related analogues (Loland et al., 2012; Mereu et al., 2017; Keighron et al., 2019) acting as potential DAT inhibitors. Followed by DAT inhibition, DA D1 receptors seem to be the key mediators in the downstream signaling process (Kalaba et al., 2017) involved in SRM. It is of note that, similar to previous findings, we failed to observe additional effects of the drug treatment on defined behavioral parameters. This speaks in favor of a specific action on memory and not on other behaviorally relevant central nervous processes.

Previous studies have shown that 30 min after a peripheral injection of *rac*-CE-123, elevated DAT and DA receptor 1 protein levels in CA1 and CA3 was produced in the hippocampus (Kristofova et al., 2018). Based on these data, it is plausible to state that *rac-*CE-123 temporarily (i.e., <6 h) protect the memory trace against retroactive interference by manipulating DA signaling in the brain. The action of the drugs cannot easily be explained by an alteration of the general social behavior of the experimental subjects (e.g., reduced interest in the 2nd sampled juvenile or increased aggressive behavior that may have covered reduced investigation) as the behavioral parameters analyzed here failed to differ between vehicle and drug treatment (Table 2). Research studies in rodents revealed that consolidation of long-term SRM is supported by information processing within defined brain areas including the olfactory bulb, anterior olfactory nucleus, medial prefrontal cortex, medial amygdala, basolateral amygdala, and different sub-regions of the hippocampus (Richter et al., 2005; Hitti and Siegelbaum, 2014; Noack et al., 2015; Tanimizu et al., 2017; Lin et al., 2018). Systemic and direct infusion of DA D1 receptor agonists either into the frontal cortex or into the nucleus accumbens improved short-term SRM in rats (Di Cara et al., 2007). Intra-insular cortex administration of agonists for DA D1/D5 receptors, β-adrenergic and serotonergic 5-HT_1A_ receptors improved the consolidation of SRM in rats (Cavalcante et al., 2017). Further, the potentiation of CA3-CA1 hippocampal synapses facilitates the consolidation of object recognition memory (Clarke et al., 2010). Due to the route of administration used in our study and the analysis of the DA levels in the nucleus accumbens only, we cannot relate the interference blocking effect of the tested drugs to an action within defined brain areas. Inspired by the fact that we could—at least as a potential target—identify the nucleus accumbens, further studies are in progress in which we will analyze the impact of our drugs on distinct areas in which the processing of information for SRM takes place in more detail. Different lines of investigation suggested a contribution of the dopaminergic system in distinct brain regions beyond the nucleus accumbens to the generation of short-term and long-term SRM of laboratory rats. Among them, the hippocampus and striatum are likely to be interesting brain areas (Garrido Zinn et al., 2016; Cavalcante et al., 2017) in which an increased DA signaling might contribute to a “stabilization” of the “SRM trace” and thereby making it resistant against interference.

The enantiomer *S*-CE-123 was able to block retroactive interference at a S_i_ of 6 h (Figure 3B), but not at a S_i_ of 3 h (Figure 3A). This indicates that *S*-CE-123 and *rac-*CE-123 administered *via* the same route and dose may affect differently the dopaminergic signaling relevant for SRM. This could be due to a different profile of washin and washout of the drugs targeting the brain tissue. Unpublished data show that compared to *rac*-CE-123, *S*-CE-123 is detectable in a ~5–10 times higher concentration in both liquor and brain tissue after intraperitoneal administration in adult male rats. The impression of a different duration of action of *S*- vs. *rac*-CE-123 is—to some aspect—supported by the microdialysis data. The release profile of the racemate at a dosage of 10 mg/kg differs from that of the *S*-enatiomer by showing elevated DA levels at the sample collected 20–40 min after treatment when *rac*-CE-123 is already indistinguishable from baseline (Figure 5B). In addition, a distinct action of the two drugs on different phases of SRM consolidation might be hypothesized: previous studies demonstrated two separate phases of sensitivity within the first 24 h after learning in paradigms testing SRM using the protein synthesis blocker anisomycin. This resulted in the hypothesis that the consolidation of long-term SRM requires two stages of protein synthesis with a gap of sensitivity to anisomycin at ~3 h after learning (Richter et al., 2005; Wanisch et al., 2008). Thus, DA signaling might be involved in the consolidation of SRM at both stages of anisomycin sensitivity. In this context, the effects of *S*-CE-123 differ to that of *rac*-CE123 in blocking the retroactive interference induced at 3 h vs. 6 h after the 1st sampling. Upon first view, this could result from a counter regulatory mechanism of the *S*-enantiomer in the administered racemate. However, such conclusions would be too premature without further studies investigating possible differences in the effects between the two CE-123 treatments including the analysis of molecular mechanisms involved.

The results of the experiment in which we administered *S*-CE-123 in the context with the induction of proactive interference failed to provide a protective effect of this enantiomer for a memory of the 2nd sampled stimulus animals (Figure 4). This speaks in favor of a specific effect of this substance on retroactive, but not proactive interference, and indicates distinct neuronal procedures underlying both phenomena. Previous studies suggested a time-depending interaction of two subsequently initiated memory traces in SRM (Engelmann, 2009), the present data suggest that DA signaling might be involved differently in the generation of retroactive and proactive interference.

Taken together, the results of the present study, show for the first time, that modafinil-derived drugs increasing extracellular DA in the nucleus accumbens and acting at both the DAT and DA D1 receptor are able to make SRM resistant against retroactive interference. The drug- and time-dependent action suggests distinct action profiles of the different drugs and provides insight into the mechanisms underlying the consolidation of SRM which requires further investigations. Further studies will focus on the cellular mechanisms *via* which the modafinil analogues tested here affect SRM. The molecular signatures linked to the blockade of interference are likely to provide further insight into the neurobiological basis of this type of learning and memory in mammals.

## Author Contributions

JC-P performed the animal experiments, treated the animals, performed a part of the statistical analysis of the behavioral data and contributed to the manuscript draft. PK was involved in the design and synthesis of the drugs; he also contributed to the design of the study. HV analyzed part of the behavioral data and provided the first draft of the manuscript. NA was involved in the synthesis of *rac*-CE-125, VD in that of *S*-CE-123. ME designed and supervised the behavioral experiments and contributed to the writing of the manuscript. GL initiated the project and contributed to the study design and to the writing of the manuscript. NS, SS and KE designed and performed the microdialysis experiments including statistical analysis and wrote the draft of the respective parts in the manuscript.

## Conflict of Interest Statement

The authors declare that the research was conducted in the absence of any commercial or financial relationships that could be construed as a potential conflict of interest.
